# Surgical Versus Non-surgical Management of Acute and Subacute Infective Endocarditis in Patients With Rheumatic Heart Disease During the COVID-19 Pandemic: A Propensity-Matched Analysis

**DOI:** 10.7759/cureus.114462

**Published:** 2026-08-13

**Authors:** Richard Nudotor, Norbert Hootsmans, Ian Bussey, Banda A Khalifa, Murtaza Dawood

**Affiliations:** 1 Surgery, Anne Arundel Medical Center, Annapolis, USA; 2 Epidemiology, Johns Hopkins Bloomberg School of Public Health, Baltimore, USA; 3 Cardiac Surgery, Anne Arundel Medical Center, Annapolis, USA

**Keywords:** adverse outcomes, covid-19 pandemic, infective endocarditis, medical-surgical, rheumatic disease

## Abstract

Background: Infective endocarditis remains a life-threatening condition requiring timely medical and surgical management. The COVID-19 pandemic posed unprecedented challenges to healthcare delivery, yet the outcomes of surgical versus conservative management among patients with infective endocarditis and underlying rheumatic heart disease during this period remain poorly defined.

Objective: This study aims to compare 30-day outcomes following surgical versus conservative management among adult patients with acute and subacute infective endocarditis in the setting of rheumatic heart disease during the COVID-19 pandemic.

Methods: Adult patients (≥18 years) admitted with acute or subacute infective endocarditis and underlying rheumatic heart disease between January 20, 2020, and July 20, 2021, were identified from the TriNetX COVID-19 Research Network. Patients with laboratory-confirmed COVID-19 infection were excluded. Patients undergoing valve surgery within one month of the index admission were compared with those managed conservatively. One-to-one propensity score matching was performed using demographic characteristics, comorbidities, laboratory values, intensive care utilization, left ventricular ejection fraction, and antibiotic therapy. The primary endpoint was 30-day all-cause mortality. Secondary outcomes included stroke, respiratory failure, pulmonary embolism, deep vein thrombosis, splenic abscess, lung abscess, and cardiac arrhythmias.

Results: A total of 573 patients met the inclusion criteria, including 45 patients who underwent surgery and 528 patients managed conservatively. After propensity score matching, 36 patients remained in each cohort with well-balanced baseline characteristics. Surgical management was associated with significantly lower 30-day mortality compared with conservative treatment (97.1% vs. 78.1% survival; *p*=0.017). Patients undergoing surgery also had lower risks of stroke (risk difference (RD) 50.0%, *p*<0.001), splenic abscess (RD 29.4%, *p*=0.001), lung abscess (RD 29.4%, *p*=0.001), and pulmonary embolism (RD 45.5%, *p*=0.001). Respiratory failure occurred more frequently in the surgical cohort (RD −47.6%, *p*=0.001), while rates of deep vein thrombosis and cardiac arrhythmias were similar between groups.

Conclusions: Among adults with acute and subacute infective endocarditis and underlying rheumatic heart disease treated during the COVID-19 pandemic, surgical management was associated with improved 30-day survival and lower rates of major embolic and infective complications compared with conservative management. These findings support the continued role of timely surgical intervention in appropriately selected patients, even during periods of significant healthcare system strain.

## Introduction

Valvular heart disease remains a major cause of cardiovascular morbidity and mortality worldwide, and its global burden is expected to increase substantially over the coming decades [1]. Rheumatic heart disease (RHD) continues to be the leading cause of valvular heart disease in many low- and middle-income countries, whereas degenerative aortic valve disease predominates in developed nations [1]. Patients with RHD are at increased risk of developing infective endocarditis (IE), a life-threatening infection of the endocardial surface of the heart that is associated with substantial morbidity and mortality. Despite advances in antimicrobial therapy and cardiac surgery, IE remains one of the most devastating valvular diseases, with reported in-hospital mortality approaching 22% and five-year mortality exceeding 45% [2]. Globally, an estimated 1.1 million new cases of infective endocarditis occurred in 2019, resulting in approximately 60,000 deaths and the loss of 1.7 million disability-adjusted life years [3]. The increasing incidence of infective endocarditis in both developed and transitioning nations has been attributed to longer life expectancy, a growing prevalence of structural heart disease, increasing use of intracardiac devices and prosthetic valves, and the ongoing epidemic of intravenous drug use [4].

The coronavirus disease 2019 (COVID-19) pandemic, caused by severe acute respiratory syndrome coronavirus 2 (SARS-CoV-2), placed unprecedented strain on healthcare systems worldwide. Following its declaration as a global pandemic by the World Health Organization in early 2020, many countries implemented public health measures that altered routine healthcare delivery. In the United States, reductions in hospital admissions, outpatient visits, diagnostic testing, and procedural volumes were observed across multiple medical specialties during the early phases of the pandemic [5,6]. Cardiovascular care was similarly affected, with reports describing reductions in cardiovascular investigations, including transesophageal echocardiography and other diagnostic procedures essential for the evaluation of infective endocarditis [6]. In addition, concerns regarding nosocomial COVID-19 transmission further complicated inpatient care during this period [7].

Timely surgical intervention remains a cornerstone in the management of appropriately selected patients with infective endocarditis, particularly those with heart failure, uncontrolled infection, or embolic complications [8]. However, limited data exist regarding the outcomes of surgical versus conservative management among patients with infective endocarditis and underlying RHD who were treated during the COVID-19 pandemic. Therefore, we compared 30-day clinical outcomes between patients undergoing surgical and conservative management for acute and subacute infective endocarditis in the setting of RHD during the COVID-19 pandemic using a propensity score-matched analysis.

## Materials and methods

Database

TriNetX is a global federated health research network that provides access to de-identified electronic medical records (EMRs), including diagnoses, procedures, medications, laboratory values, and other clinical data from participating healthcare organizations (HCOs). The platform is compliant with the Health Insurance Portability and Accountability Act (HIPAA) and contains the TriNetX COVID-19 Research Network, one of the largest multicenter datasets of patients treated during the COVID-19 pandemic. This study utilized data from 89 participating HCOs, comprising more than 102 million de-identified patient records, queried in June 2023. The use of de-identified data exempted the study from informed consent requirements. The study was approved by the Institutional Review Board of Anne Arundel Medical Center.

Cohort creation

Adult patients (≥18 years) admitted between January 20, 2020, and July 20, 2021, with acute or subacute infective endocarditis in the setting of underlying RHD were identified from the TriNetX COVID-19 Research Network. Infective endocarditis was identified using ICD-10 diagnostic codes for acute and subacute infective endocarditis, while RHD was identified using ICD-10 codes for rheumatic mitral, aortic, and tricuspid valve disease. The complete list of ICD-10 codes used for cohort identification is provided in Appendix A. Patients with laboratory-confirmed SARS-CoV-2 infection and those younger than 18 years were excluded.

**Figure 1:**
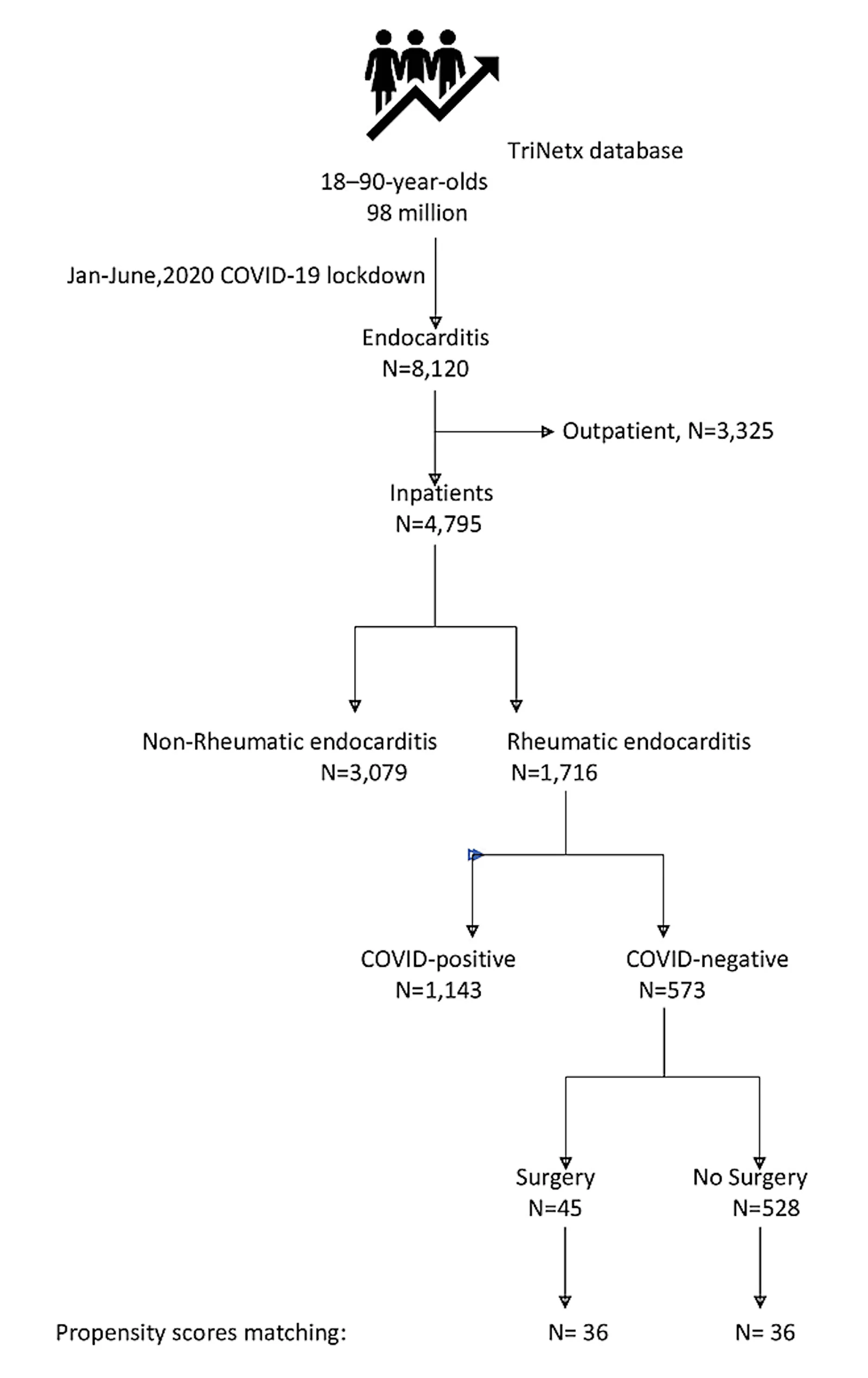
Distribution of patients in surgery and non-surgery cohorts.

Two treatment cohorts were created according to management strategy. The surgical cohort included patients who underwent their first qualifying valve procedure within 30 days of the index hospitalization for infective endocarditis. Restricting analysis to the first qualifying procedure minimized inclusion of repeat admissions and duplicate procedures. Surgical procedures were identified using CPT codes for mitral valve replacement (33430, 33425, 33426, 33427), transcatheter mitral valve repair (33418, 33419), tricuspid valve repair (33464, 33465), surgical aortic valve replacement (33405), and transcatheter aortic valve replacement (33361). The conservative management cohort included patients who did not undergo valve surgery within 30 days of the index hospitalization (Figure 1).

Variables and outcomes

The primary outcome was 30-day all-cause mortality following the index hospitalization. Secondary outcomes included stroke, respiratory failure, deep vein thrombosis, pulmonary embolism, splenic abscess, lung abscess, and cardiac arrhythmia occurring within 30 days. Demographic characteristics, comorbidities, laboratory values, procedural information, and outcome variables were extracted using ICD-10 diagnosis codes, CPT procedure codes, and Logical Observation Identifiers Names and Codes (LOINC). Baseline variables included age, sex, race, ethnicity, atrial fibrillation/flutter, heart failure, chronic obstructive pulmonary disease, hypertension, ischemic heart disease, chronic kidney disease, peripheral vascular disease, cerebrovascular disease, diabetes mellitus, overweight/obesity, prior coronary angioplasty, malnutrition, tobacco use, alcohol-related disorders, serum creatinine, bilirubin, international normalized ratio (INR), intensive care unit admission, left ventricular ejection fraction, and antibiotic therapy. Antibiotic regimens for both treatment groups are summarized in Table 1.

**Table 1:** Matched medications used by patients in the cohorts A p-value < 0.05 is statistically significant.

Medication	Surgery	No Surgery	P-value
Penicillin and beta-lactams	27 (51.9%)	32 (51.6%)	0.341
Aminoglycoside and quinolones	10 (19.2%)	13 (21.0%)	0.201
Macrolides and others	15 (28.9%)	17 (27.4%)	0.552

Propensity score matching

To compare outcomes between patients undergoing surgical and conservative management while minimizing treatment-selection bias, propensity score matching was performed before outcome analysis. Propensity scores were estimated using a logistic regression model implemented within the TriNetX Analytics platform. Variables included in the propensity score model were age, sex, race, ethnicity, atrial fibrillation/flutter, heart failure, chronic obstructive pulmonary disease, hypertension, ischemic heart disease, chronic kidney disease, peripheral vascular disease, cerebrovascular disease, diabetes mellitus, overweight/obesity, prior coronary angioplasty, malnutrition, tobacco use, alcohol-related disorders, serum creatinine, bilirubin, INR, intensive care unit admission, left ventricular ejection fraction, and antibiotic therapy.

**Figure 2:**
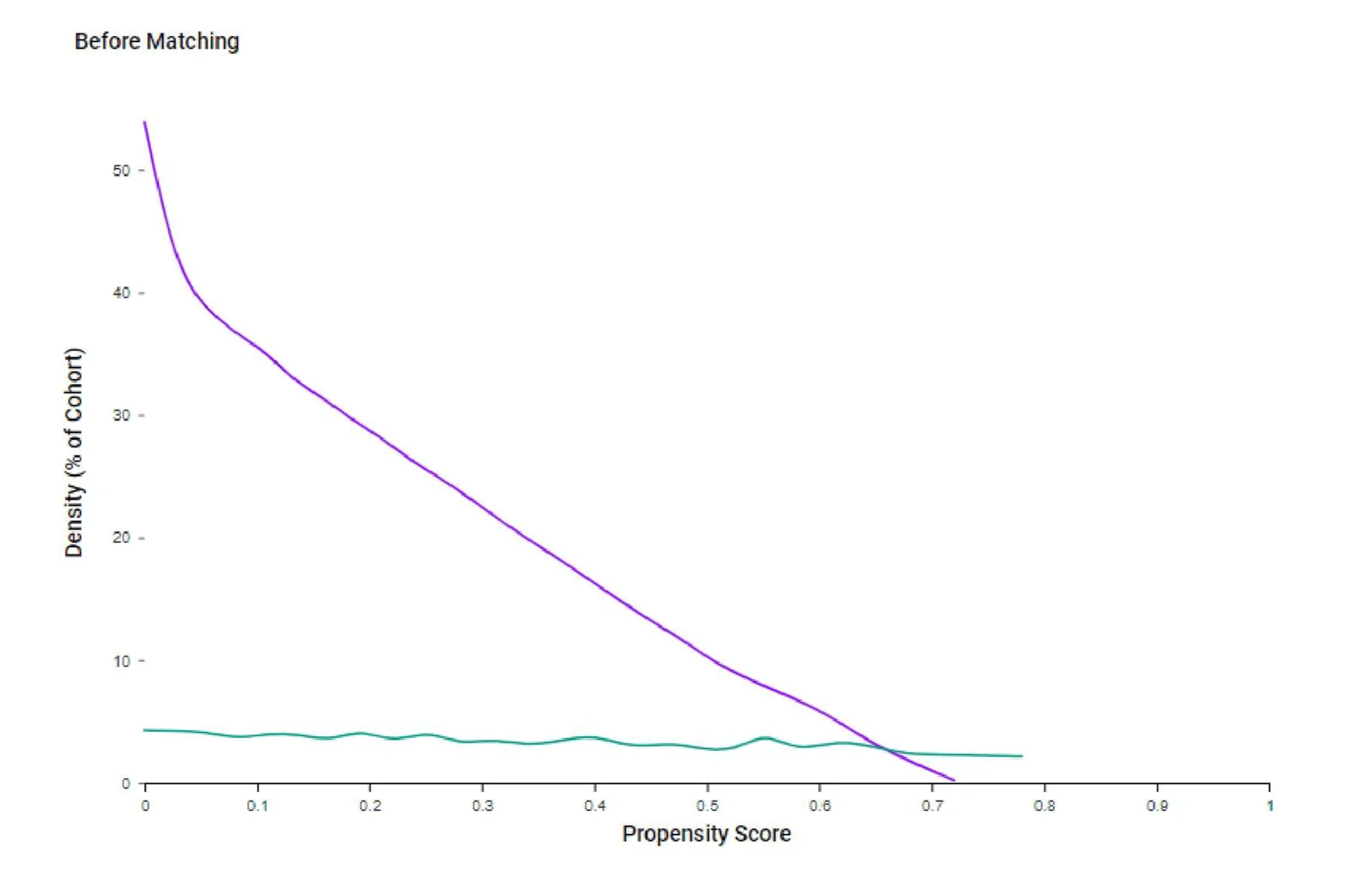
Propensity score prior to matching on demographic and clinical characteristics. The purple line indicates the no surgery group, and the green line indicates the surgery group.

**Figure 3:**
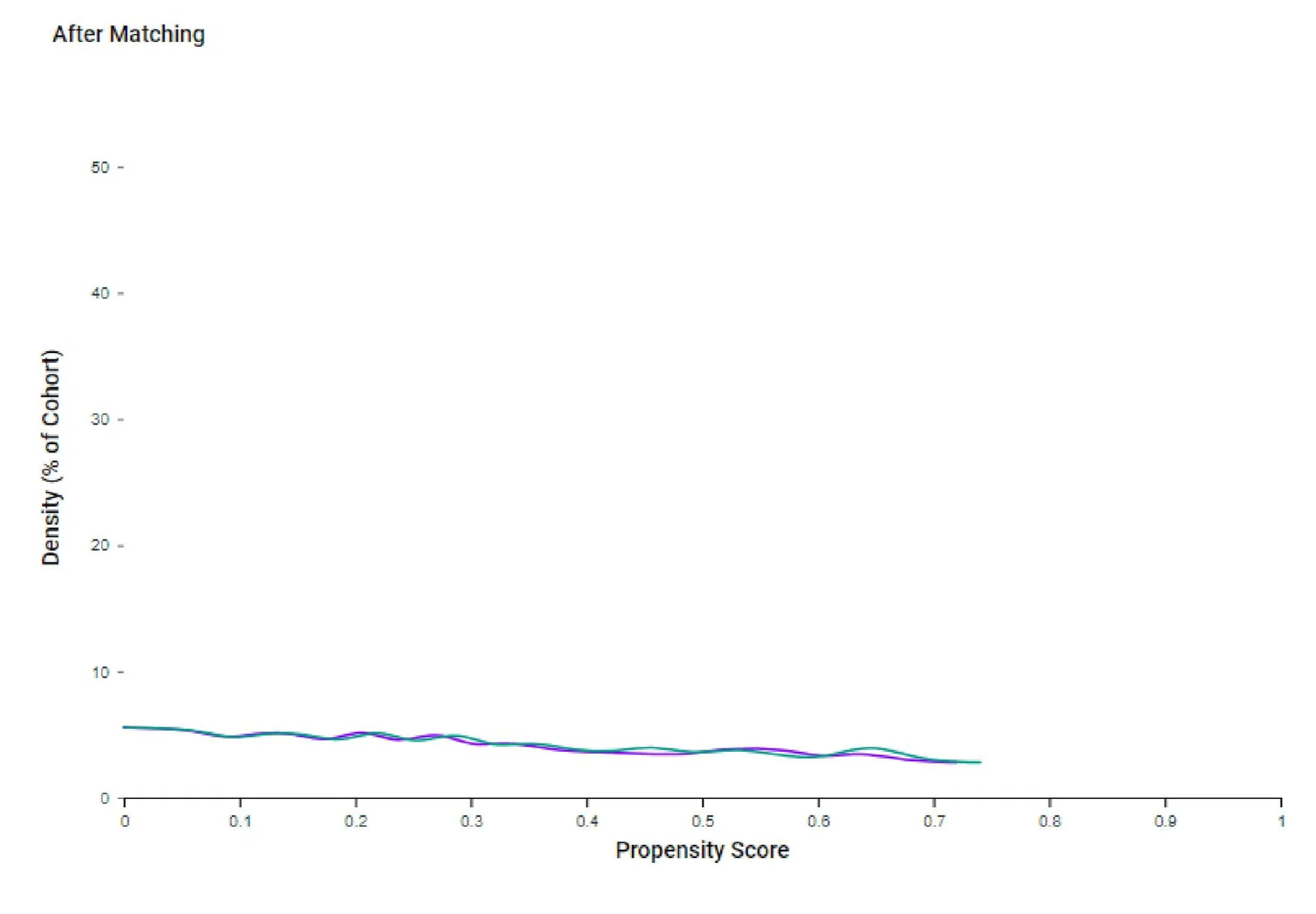
Propensity score after matching demographic and clinical characteristics. The purple line indicates the no surgery group, and the green line indicates the surgery group.

Patients were matched in a 1:1 nearest-neighbor fashion without replacement, pairing each surgical patient with the non-surgical patient having the closest propensity score. Covariate balance between the matched cohorts was assessed using standardized comparisons within the TriNetX platform, and baseline characteristics after matching are presented in the next section. Propensity score distributions before and after matching are shown in Figures 2, 3. After matching, the final study population consisted of 36 patients in the surgical cohort and 36 patients in the conservative management cohort, who were subsequently compared for 30-day clinical outcomes.

Statistical analysis

All statistical analyses were performed using the TriNetX Analytics platform. Baseline demographic and clinical characteristics were summarized for the matched cohorts, with categorical variables reported as frequencies and percentages and continuous variables as means with standard deviations. Comparisons between matched cohorts were performed using the statistical methods implemented within the TriNetX platform.

**Figure 4:**
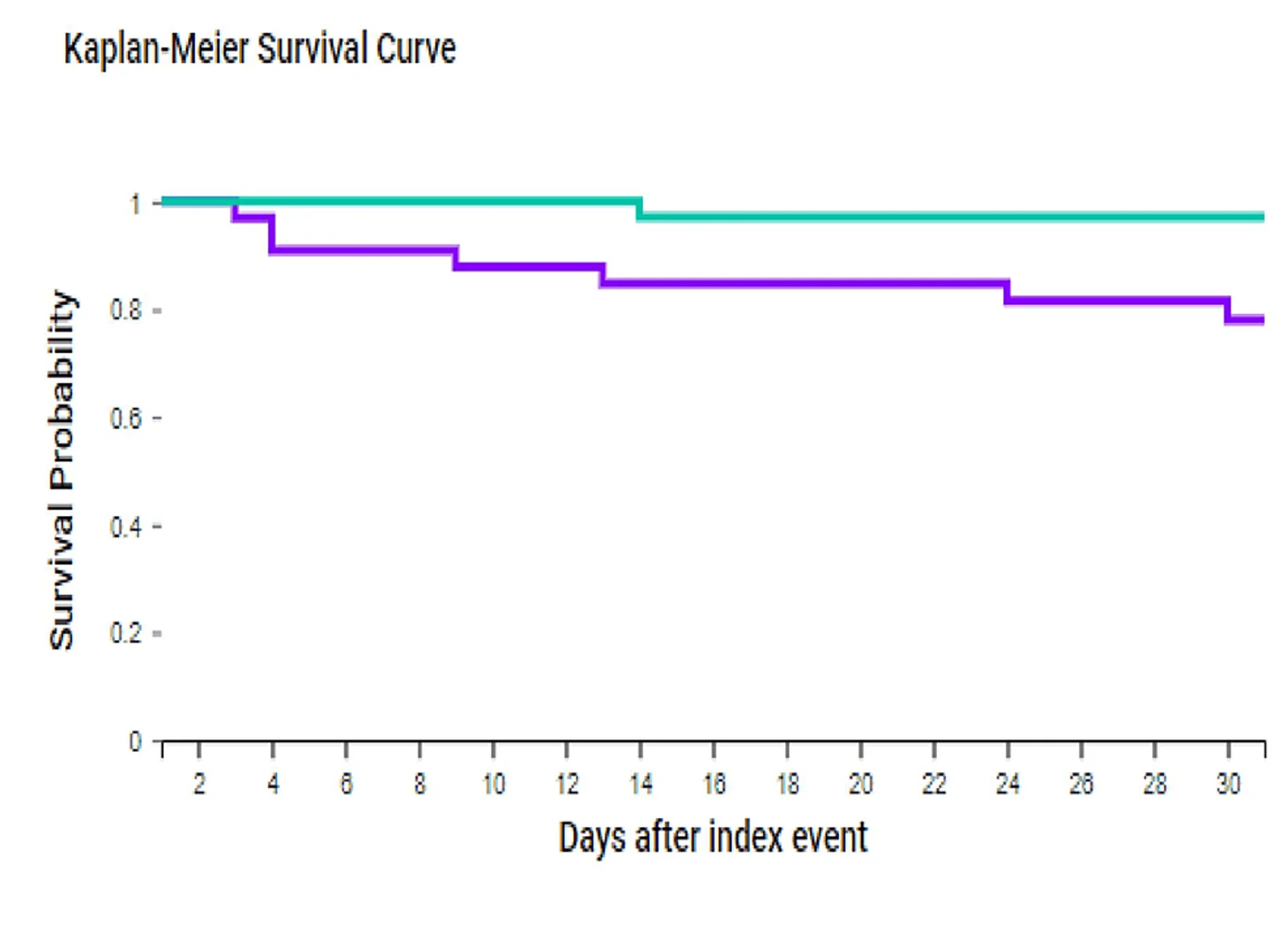
Survival probability of two cohorts after matched analysis. The purple line indicates the no surgery group, and the green line indicates the surgery group.

The primary outcome, 30-day all-cause mortality, was evaluated using Cox proportional hazards regression analysis. Survival distributions between the surgical and non-surgical cohorts were compared using the log-rank test and visualized with Kaplan-Meier survival curves, as shown in Figure 4.

Secondary outcomes occurring within 30 days of the index hospitalization were reported as risks, risk differences (RDs), risk ratios (RRs), odds ratios (ORs), and corresponding 95% confidence intervals. RD was defined as the absolute difference in event rates between the two treatment groups. All statistical tests were two-sided, and statistical significance was defined as a p-value <0.05.

## Results

Baseline characteristics of the study subjects

The baseline demographic and clinical characteristics of the surgery and no surgery groups before propensity score matching are presented in Table 2. Compared with the no surgery group, patients in the surgery group were significantly younger (p=0.023) and were more likely to be Black (p=0.005), have atrial fibrillation/flutter (p=0.0001), malnutrition (p<0.001), peripheral vascular disease (p<0.001), cerebrovascular disease (p<0.001), prior coronary angioplasty with implant or graft (p<0.001), and intensive care unit admission (p<0.001). The most commonly performed valvular procedure was mitral valve replacement (18 patients, 40%), and penicillin was the most frequently administered antibiotic (p=0.001).

**Table 2:** Baseline characteristics of the patients in the cohort prior to matching SD: standard deviation, COPD: chronic obstructive pulmonary disease, PCI: percutaneous coronary intervention, INR: international normalized ratio, ICU: intensive care unit, LVEF: left ventricular ejection fraction.

Codes	Characteristics	No Surgery	Surgery	P-value
		N = 528	N = 45	
AI	Age at index	-	-	-
-	Mean +/- SD	53.2 +/- 18.6	46.7 +/- 16.2	0.023
-	Gender	-	-	-
M	Male	308 (58.3%)	25 (55.6%)	0.717
F	Female	220 (41.7%)	20 (44.4%)	-
-	Race	-	-	-
2106-3	White	418 (79.2%)	35 (77.8%)	0.826
2054-5	Black	48 (9.1%)	10 (22.2%)	-
-	Ethnicity	-	-	-
2135-2	Hispanic	63(11.9%)	10 (22.2%)	0.047
2186-5	Non-hispanic	382 (72.3%)	35 (78.8%)	-
J40 -J47	COPD	85 (16.1%)	10 (22.2%)	0.289
I73	Peripheral vascular disease	30 (5.7%)	10 (22.2%)	<0.001
I60-I69	Cerebrovascular disease	96 (18.2%)	22 (48.9%)	<0.001
Z95.5	Previous PCI	15(2.8%)	10 (22.2%)	<0.001
Z95.2	Presence of prosthetic valve	61(11.6%)	16 (35.6%)	<0.001
I48	Atrial fibrillation or flutter	97 (18.4%)	18 (40%)	0.001
I10	Essential hypertension	133 (25.2%)	16 (35.6%)	0.128
E08 - E13	Diabetes mellitus	111 (21.0%)	11 (24.4%)	0.59
I50	Heart failure	122 (23.1%)	18 (40%)	0.011
I20-I25	Ischemic heart disease	115 (21.8%)	20 (44.4%)	0.001
N18	Chronic kidney disease	82 (15.5%)	13(28.9%)	0.021
E65-E68	Overweight and obesity	45 (8.5%)	10 (22.2%)	0.003
E40 - E46	Malnutrition	36(6.8%)	11 (24.4%)	<0.001
Z72.0	Tobacco use	39(7.4%)	10 (22.2%)	0.001
F10	Alcohol-related disorder	26(4.9%)	10 (22.2%)	<0.001
9024	Mean Creatinine +/- SD (mg/dL)	1.4 +/- 1.7	1.3 +/- 1.5	0.77
9048	Mean Bilirubin +/- SD (mg/dL)	0.9 +/- 2.2	1.0 +/- 1.2	0.981
9032	Mean INR +/- SD	2.1 +/- 3.5	1.3 +/- 0.4	0.17
1014309	ICU care	81 (15.3%)	17 (37.8%)	<0.001
2003	LVEF	-	-	-
-	<30%	10 (1.9%)	10 (22.2%)	<0.001
-	30–50%	13 (2.5%)	0 (0%)	0.287
-	>50%	39 (7.4%)	10 (22.2%)	0.001

Following 1:1 propensity score matching, 36 patients remained in each cohort. After matching, no statistically significant differences were observed in the baseline demographic or clinical characteristics between the surgery and no surgery groups (Table 3), indicating successful covariate balance between the matched cohorts.

**Table 3:** Baseline demographic and clinical characteristics of cohort after matching SD: standard deviation, COPD: chronic obstructive pulmonary disease, PCI: percutaneous coronary intervention, INR: international normalized ratio, ICU: intensive care unit, LVEF: left ventricular ejection fraction.

Codes	Characteristics	No Surgery	Surgery	P-value
		N = 36	N = 36	
AI	Age at Index	-	-	-
-	Mean +/- SD	46.4 +/- 18.1	46.0 +/- 15.2	0.91
-	Gender	-	-	-
M	Male	19 (52.8%)	21 (58.3%)	0.635
F	Female	17 (47.2%)	15 (41.7%)	
-	Race	-	-	-
2106-3	White	28 (77.8%)	27 (75%)	0.781
2054-5	Black	8 (22.2%)	9 (25%)	-
-	Ethnicity	-	-	-
2135-2	Hispanic	10 (27.8%)	10 (27.8%)	0.772
2186-5	Non-Hispanic	29 (80.6%)	28 (77.8%)	-
J40-J47	COPD	10 (27.8%)	10 (27.8%)	1
I73	Peripheral vascular disease	10 (27.8%)	10 (27.8%)	1
I60-I69	Cerebrovascular disease	14 (38.9%)	15 (41.7%)	0.81
Z95.5	Previous PCI	0 (0%)	10 (27.8%)	0.001
Z95.2	Presence of prosthetic valve	10 (27.8%)	14 (38.9%)	0.317
I48	Atrial fibrillation or flutter	14 (38.9%)	12 (33.3%)	0.624
I10	Essential Hypertension	11 (30.6%)	14 (38.9%)	0.458
E08 - E13	Diabetes mellitus	10 (27.8%)	10 (27.8%)	1
I50	Heart failure	13 (36.1%)	15 (41.7%)	0.629
I20-I25	Ischemic heart disease	12 (33.3%)	14 (38.9%)	0.624
N18	Chronic kidney disease	11 (30.6%)	10 (27.8%)	0.795
E65-68	Overweight and obesity	10 (27.8%)	10 (27.8%)	1
E40 - E46	Malnutrition	10 (27.8%)	10 (27.8%)	1
Z72.0	Tobacco use	10 (27.8%)	10 (27.8%)	1
F10	Alcohol-related disorder	10 (27.8%)	10 (27.8%)	1
9024	Mean Creatinine +/- SD (mg/dL)	1.0 +/- 0.8	1.4 +/- 1.6	0.323
9048	Mean Bilirubin +/- SD (mg/dL)	0.8 +/- 1.0	0.9 +/- 0.9	0.939
9032	Mean INR +/- SD	3.4 +/- 7.5	1.3 +/- 0.4	0.147
1014309	ICU care	15 (41.7%)	12 (33.3%)	0.465
2003	LVEF	-	-	-
-	<30%	10 (27.8%)	10 (27.8%)	<0.001
-	30–50%	0	0	-
-	>50%	10 (27.8%)	10 (27.8%)	-

Risk of adverse outcomes

After matched analysis, there was a higher risk of 30-day mortality among patients who did not undergo surgery compared to those who did, with a survival probability of 78.1% and 97.1%, respectively (p=0.017), as shown in Figure 2. As shown in Table 4, patients who underwent surgery were less likely to develop stroke (RD: 50%, 95% CI: 28.1-71.9, p<0.001), splenic abscess (RD: 29.4%, 95% CI: 14.1-44.7, p=0.001), lung abscess (RD: 29.4%, 95% CI: 14.1-44.7, p=0.001), and pulmonary embolism (RD: 45.5%, 95% CI: 24.6-66.3, p=001). However, patients who underwent surgery had a statistically significant risk of respiratory failure (RD: -47.6%, 95% CI: -69.0 to -26.3, p=0.001). There was no difference in risk of acute deep vein thrombosis (RR: 1.14, 95% CI: 0.69-2.85, p=0.355) and cardiac arrhythmia (RR: 0.73, 95% CI: 0.40-1.32) between the two cohorts within the 30-day period.

**Table 4:** Matched analysis of 30-day mortality and other adverse outcomes among patients in the cohort A p-value < 0.05 is statistically significant. SD: standard deviation, OR: odds ratio, RD: risk reduction, CI: confidence interval.

Adverse Outcomes	No Surgery	Surgery	RD (95% CI)	RR (95% CI)	OR (95% CI)	p-value
Stroke	50.00%	0.00%	50% (28.1, 71.9)	-	-	<0.001
Respiratory failure	0.00%	47.60%	-47.6% (-69.0, -26.3)	-	-	0.001
Deep vein thrombosis	40.00%	28.60%	11.4% (-12.9, 35.8)	1.40 (0.69, 28.5)	1.67 (0.56, 4.93)	0.355
Splenic abscess	29.40%	0.00%	29.4% (14.1, 44.7)	-	-	<0.001
Lung abscess	29.40%	0.00%	29.4% (14.1, 44.7)	-	-	0.001
Pulmonary embolism	45.50%	0.00%	45.5% (24.6, 66.3)	-	-	0.001
Cardiac arrhythmias	45.40%	62.50%	-17.0% (-48.6, 14.5)	0.73 (0.40, 1.32)	0.50 (0.13, 1.86)	0.299

## Discussion

In this propensity score-matched analysis of adult patients with acute and subacute infective endocarditis in the setting of RHD who were treated during the COVID-19 pandemic, patients who underwent surgery had significantly better 30-day outcomes than those managed without surgery. Specifically, the surgery group demonstrated higher 30-day survival and lower risks of stroke, splenic abscess, lung abscess, and pulmonary embolism. These findings are consistent with contemporary guideline recommendations supporting early surgical intervention in appropriately selected patients with infective endocarditis to control infection, prevent embolic complications, and preserve cardiac function when medical therapy alone is insufficient [8]. Although postoperative respiratory failure occurred more frequently among patients undergoing surgery, the overall clinical benefit favored operative management.

The improved outcomes observed in the surgery group are consistent with the natural history and pathophysiology of infective endocarditis. Persistent infection, progressive valvular destruction, and systemic embolization are major determinants of adverse outcomes when definitive source control is not achieved [2]. Surgical intervention removes infected tissue and corrects structural valvular abnormalities, thereby reducing the risk of persistent infection and embolic complications [8]. Consistent with these mechanisms, patients in the surgery group experienced significantly lower rates of stroke, pulmonary embolism, splenic abscess, and lung abscess. Conversely, the higher mortality observed in the no surgery group may reflect ongoing infection and progressive valvular dysfunction despite antimicrobial therapy, although residual differences in disease severity between the groups cannot be excluded.

Although this study was conducted during the COVID-19 pandemic, the pandemic should be regarded as the clinical context rather than the primary exposure under investigation. Previous studies have demonstrated substantial reductions in hospital presentations, cardiovascular diagnostic testing, procedural volumes, and access to cardiovascular services during the pandemic [5,6,9-12]. Such disruptions raised concerns regarding timely diagnosis and definitive treatment of patients with serious cardiovascular diseases, including infective endocarditis. However, our analysis was not designed to quantify healthcare delays, procedural deferrals, or reductions in service availability. Rather, our findings demonstrate that among patients treated during this period, surgery remained associated with significantly better short-term outcomes than no surgery, emphasizing the importance of maintaining timely access to cardiac surgical care during future healthcare emergencies.

The higher incidence of postoperative respiratory failure observed in the surgery group deserves consideration. Cardiac valve surgery frequently requires prolonged cardiopulmonary bypass, postoperative mechanical ventilation, and intensive care support, all of which contribute to postoperative pulmonary complications. Prolonged mechanical ventilation and perioperative factors have been identified as important predictors of respiratory failure following cardiac surgery [13]. Nevertheless, despite this increased postoperative morbidity, patients undergoing surgery experienced substantially better survival and fewer major infective and embolic complications, suggesting that the overall benefits of surgery outweighed these short-term risks.

The strengths of this study include the use of a large multicenter electronic health record database representing more than 98 million patients across multiple healthcare organizations and the application of propensity score matching to reduce baseline differences between the surgery and no surgery groups. Matching on demographic characteristics, comorbidities, laboratory parameters, intensive care utilization, left ventricular ejection fraction, and antibiotic therapy strengthened the validity of the comparative analyses and reduced treatment-selection bias.

Several limitations should be acknowledged. First, this was a retrospective observational study and therefore remains susceptible to residual confounding despite propensity score matching. Second, the TriNetX database lacks detailed clinical information regarding vegetation size, microbiological etiology, echocardiographic findings, surgical indications, timing of symptom onset, timing of operative intervention, and duration of antimicrobial therapy, all of which may influence treatment decisions and outcomes. Third, although the study was conducted during the COVID-19 pandemic, healthcare utilization measures such as delays in presentation, procedural deferrals, resource limitations, and changes in institutional practice were not directly captured. Consequently, our findings should not be interpreted as demonstrating that pandemic-related healthcare disruptions caused the observed differences in outcomes. Fourth, because patients were not randomized, residual confounding by indication cannot be excluded, and unmeasured differences in disease severity may have influenced treatment selection. Finally, the relatively small number of patients in the surgery group after propensity score matching may have limited statistical power and the generalizability of our findings to other healthcare systems.

## Conclusions

Among adults with acute and subacute infective endocarditis occurring in the setting of RHD who were treated during the COVID-19 pandemic, surgery was associated with significantly higher 30-day survival and lower rates of major embolic and infective complications compared with no surgery. Although postoperative respiratory failure occurred more frequently in the surgery group, the overall findings support the continued role of timely surgical intervention in appropriately selected patients with infective endocarditis. Maintaining access to specialized cardiac surgical services during periods of healthcare system stress remains important. Future prospective studies are warranted to better define patient selection, optimal timing of surgery, and long-term outcomes in this high-risk population.

## Figures and Tables

**Figure 5:**
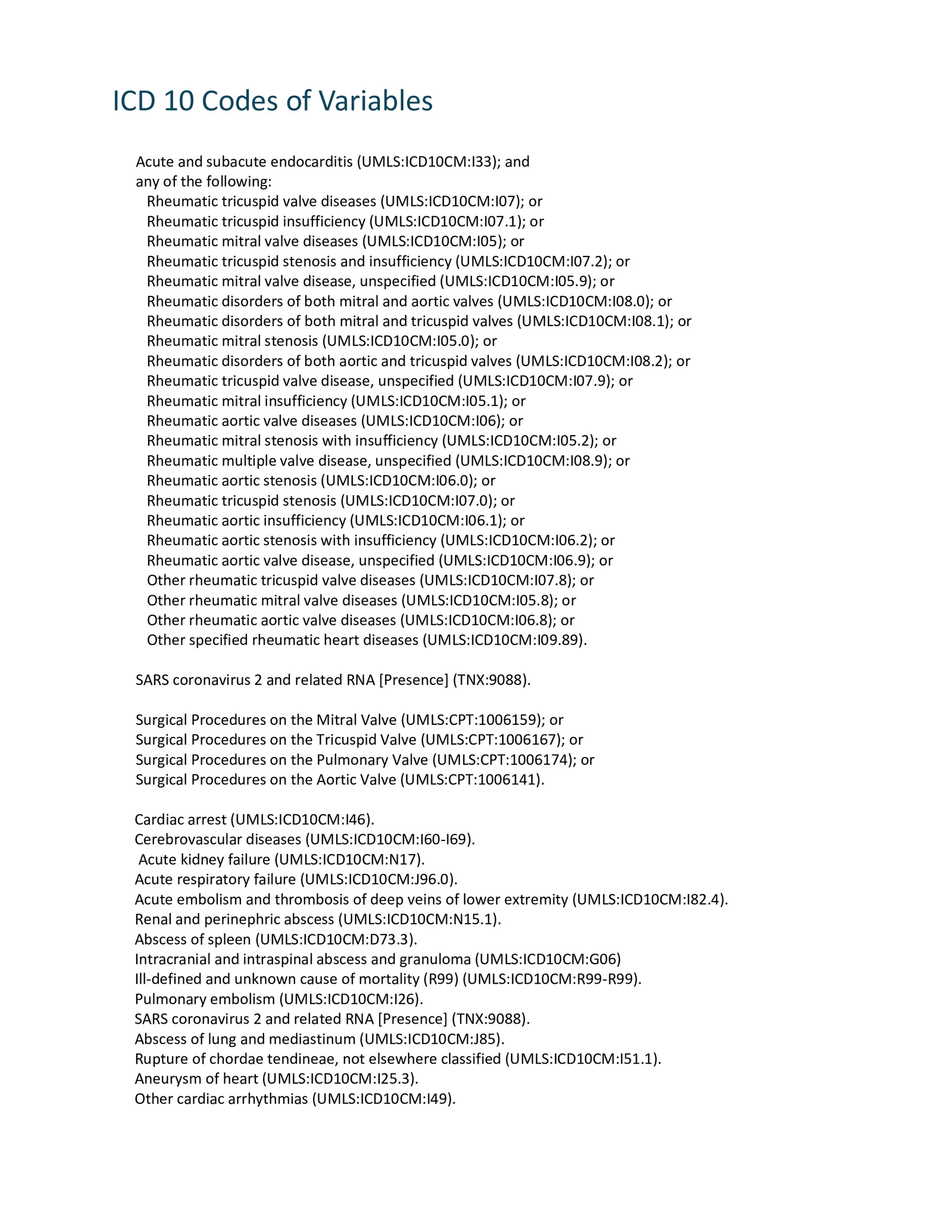
ICD-10 codes used for cohort creation and analysis ICD: International Classification of Diseases

## Appendices

Appendix A
